# An African Canine Trypanosomosis Case Import: Is There a Possibility of Creating a Secondary Focus of *Trypanosoma congolense* Infection in France?

**DOI:** 10.3390/pathogens9090709

**Published:** 2020-08-27

**Authors:** Florence Calvet, Hacène Medkour, Oleg Mediannikov, Caroline Girardet, Antoine Jacob, Mickaël Boni, Bernard Davoust

**Affiliations:** 1 1^er^ Groupe Vétérinaire, Service de Santé des Armées, 83800 Toulon, France; florence1.calvet@intradef.gouv.fr (F.C.); antoinebcj62@gmail.com (A.J.); mickael.boni@intradef.gouv.fr (M.B.); 2 IRD, AP-HM, MEPHI, Aix Marseille University, 19-21, Bd Jean Moulin, 13005 Marseille, France; hacenevet1990@yahoo.fr (H.M.); olegusss1@gmail.com (O.M.); 3 IHU Méditerranée Infection, 19-21, Bd Jean Moulin, 13005 Marseille, France; 4 22^e^ Groupe Vétérinaire, Service de Santé des Armées, 33038 Bordeaux, France; caroline.girardet@intradef.gouv.fr; 5 Institut de Recherche Biomédicale des Armées, 91220 Brétigny-sur-Orge, France; 6 Groupe de Travail en Épidémiologie Animale du Service de Santé des Armées, 13014 Marseille, France

**Keywords:** canine African trypanosomosis, military working dog, transboundary animal diseases, *Trypanosoma congolense* (forest type)

## Abstract

African animal trypanosomosis are parasitic diseases caused by several protozoa of the genus *Trypanosoma*, transmitted by hematophagous insects, essentially tsetse flies, but also, less frequently by Tabanidae and Stomoxidae. They are geolocated in a part of the continent and affect livestock animals and carnivores; dogs are especially sensitive to them. They do not seem to present a zoonotic risk. Despite the chemical prevention with trypanocides for French military working dogs on mission in Côte d’Ivoire, a fatal case induced by *Trypanosoma congolense* in France after returning from Abidjan raises the question of an imported secondary focus. The clinical case was developed and the causative agent was confirmed by microscopy and PCR methods. The three necessary pillars to create a secondary potential focus are present: the parasite introduction in a new territory, the presence and the propagation vectors, and their proximity with sensitive species.

## 1. Introduction

Animal African trypanosomosis is a group of diseases caused by several flagellated protozoa from the genus *Trypanosoma*, transmitted by hematophagous insects, essentially tsetse flies, *Glossina* genus, but also by Tabanidae and Stomoxidae, even less frequently [1,2]. Tsetse flies are the true vectors of trypanosomes. They behave like intermediate hosts for the parasite, which are essential for their biological cycle. Tabanidae and Stomoxidae are simple mechanical vectors (simple regurgitation of the parasite) [3,4]. They affect various mammal species with an acute incidence in livestock and equids. The causative agents are mainly *Trypanosoma congolense*, *Trypanosoma vivax* and *Trypanosoma evansi* [2,5]. Dogs are also susceptible to infection [6,7,8,9,10].

The human African trypanosomiasis (or sleeping sickness) is caused by strictly human parasites (*T. brucei*) but presents vectorial similarities with animal trypanosomosis [11]. It occurs in areas where tsetse flies are present (especially *Glossina morsitans*, *Glossina tachinoïdes* and *Glossina palpalis*): in vegetation, by the rivers and lakes, in forest galleries and in shrubby savannah [12]. This distribution is located between two imaginary lines: the first, from the 14th to the 10th north parallel (Senegal/ Somalia) and the second in the 20th south parallel, north of the Kalahari Desert. This area includes about 10 million square kilometers and covers 37 countries [11]. In Côte d’Ivoire, an exceptional human case of *T. brucei* and *T. congolense* coinfection has been described [13].

Animal trypanosomosis are classic acute or chronic diseases that cause fever and are accompanied by anemia, oedemas, lacrimation, lymphatic nodes hypertrophy, abortions, reduced fertility, loss of appetite and weight loss, leading to premature death in acute forms or to digestive and/or nervous signs with emaciation and subsequent death in chronic forms. Eye damage is not rare, such as keratitis, conjunctivitis and corneal clouding. These non-specific symptoms make it difficult to diagnose these diseases, for which no vaccine currently exists [8,9,10,14,15].

Canine African trypanosomosis (CAT) due to *Trypanosoma congolense* is described in Côte d’Ivoire, especially in slaughterhouses [6]. Cattle are the common hosts and the tsetse fly is the main vector. The French military kennel at Port-Bouët, which has potential breeding sites for *Glossina* spp. in its forested area, is likely to be exposed to this disease. As a precaution, since 2004, a chemoprevention with isometamidium chloride has been established, for all the military working dogs (MWD) spending time in this kennel, with Trypamidium^®^ (Boehringer Ingelheim, Lyon, France), one injection of 1 mg/kg every two months [16,17]. A recent imported case of canine trypanosomosis due to *T. congolense*, on return from the Abidjan region, raised the question of the potential for the creation of a secondary outbreak in France. In this report, we describe the clinical case and evaluate this risk of importation.

## 2. Case-Report

### 2.1. Commemoratives and Clinical Description

A seven-year-old male Belgian shepherd was based in Côte d’Ivoire from 17 December 2018 to 7 January 2019. From December 18, an injection of Trypamidium^®^ was administered, in addition to classical MWD prophylactic treatments used in tropical regions, such as collars of deltamethrin, Scalibor^®^ (MSD Santé Animale, Beaucouze, France), during the mission. On 12 February, it was rushed to the 1st Veterinary Group of the French Army Health Service (Toulon) for convulsions that had appeared suddenly two hours earlier. It was in lateral decubitus, coma, polypnea-tachypnea and its mucous membranes were congested. It also presented a bilateral mydriasis, a flexible abdomen, a temperature of 39.5 °C and a cardiac frequency of 140 bpm. The dog received a treatment against shock and a constant dose-perfusion of analgesic. The coma intensified (eyes rolled back, increased hyperventilation), and the dog was referred to the veterinary clinic Olliolis in Ollioules (Var, France). Additional examinations were performed: medical imaging was normal (scanner, chest radiography, abdominal echography). The blood count showed an anemia, leucopenia, thrombocytopenia, and biochemical analysis showed severe hypoglycemia, moderate hypoalbuminemia and uremia; alkaline phosphatase and alanine transaminase concentrations were increased.

Blood smear microscopic examination, after May–Grünwald Giemsa coloration and observation with oil-immersion-objective (×100), highlighted many nucleated elongated shapes. The blood smear also showed an anisocytosis and non-normochromic erythrocytes.

The dog died 12 h later without regaining consciousness and without further convulsions. The necropsy was carried out 15 h later on all organs including an opening of the skull and observation of the encephalon. No lesions were detected, with the exception of splenomegaly. The cytology on splenic punctures revealed a reactional spleen with a major lympho-plasma cell hyperplasia. Blood, spleen, liver, kidneys and encephalon samples were sent to the Institut Hospitalo-Universitaire Méditerranée Infection of Marseille (France) for further analyses.

### 2.2. Microscopic Observation

**Figure 1:**
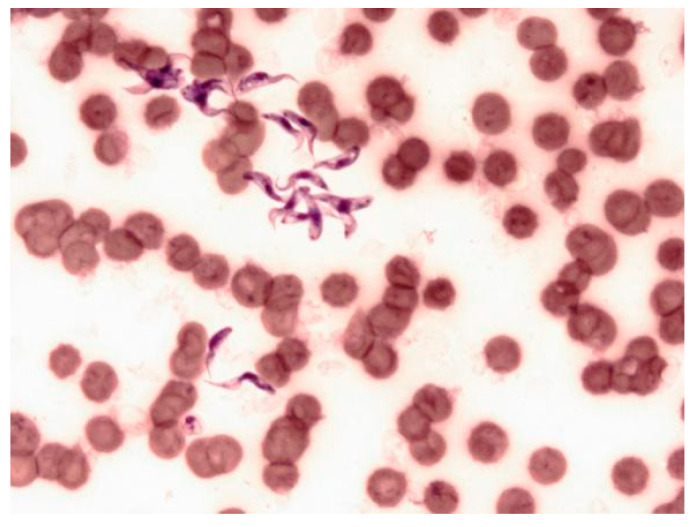
Microscopic observation of trypanosomes after Giemsa staining (G 10 × 100). The number of trypanosomes (*Trypanosoma congolense*) was estimated to be about one million parasites/mL of blood.

Blood smears were performed and after fixation in methanol; they were stained in eosin for 3 s and in methylene blue for 6 s. The slides were washed twice in a buffer and observed microscopically with an objective (×100). Microscopic observation revealed numerous trypanosomes (Figure 1).

### 2.3. Molecular Assays

DNA was extracted from 200 µL of blood and approximately 20 mg from the spleen, liver, kidney and brain samples after digestion with glass powder and proteinase K (10 µL) at 56 °C overnight. Extraction was performed on BIOROBOT EZ1 (Qiagen, Qiagen, Courtaboeuf, France), using a commercial DNA extraction kit (QIAamp DNA Mini Kit^®^, Qiagen, Courtaboeuf, France) following the manufacturer’s instructions. DNA was eluted in 200 µL. All DNA were tested by a real-time PCR (qPCR) assay targeting the 5.8S rRNA gene for *Trypanosoma* spp., with primers F5.8S_Tryp_CAACGTGTCGCGATGGATGA and F5.8S_Tryp_ ATTCTGCAATTGATACCACTTATC and probe S5.8S_Tryp_FAM-GTTGAAGAACGCAGCAAAGGCGAT. All samples were subjected to a conventional PCR targeting ~550 bps of the 28S RNA gene of Kinetoplastida parasites and sequencing by using primers: F2_ ACCAAGGAGTCAAACAGACG and R1_ GACGCCACATATCCCTAAG [18,19]. The qPCR assay was prepared in a final volume of 20 μL as previously described [19]. Amplification was performed in a CFX96 Real-Time system (BioRad Laboratories, Foster City, CA, USA) according to the following Roche protocol: an incubation step at 50 °C for two minutes and an initial denaturation step at 95 °C for five minutes, followed by 40 cycles of denaturation at 95 °C for 5 s and annealing-extension at 60 °C for 30 s. DNA of *Trypanosoma brucei* and master mixture were added as positive control and negative control, respectively. Samples were considered positive when the cycle threshold (C_t_) was lower than 35 C_t_.

PCR amplifications were performed in a Peltier PTC-200 model thermal cycler (MJ Research Inc., Watertown, MA, USA). Reaction mixtures were prepared in 50 µL volume as previously described [18]. The thermal cycling protocols were as follows: incubation step at 95 °C for 15 min, 40 cycles of one minute at 95 °C, 30 s at 57 °C and one minute at 72 °C and a final extension step for five minutes at 72 °C. All amplicons were visualized in electrophoresis on 2% agarose gels. Amplicons were then purified using NucleoFast 96 PCR plates (Macherey Nagel EURL, Hoerdt, France) according to the manufacturer’s instructions and were then sequenced using the Big Dye Terminator Cycle Sequencing Kit (Perkin Elmer Applied Biosystems, Foster City, CA, USA) with an ABI automated sequencer (Applied Biosystems). The obtained electropherograms were assembled and edited using ChromasPro software (ChromasPro 1.7, Technelysium Pty Ltd., Tewantin, Australia) and compared with those available in the GenBank database by National Center for Biotechnology Information (NCBI) BLAST (https://blast.ncbi.nlm.nih.gov/Blast.cgi).

**Figure 2:**
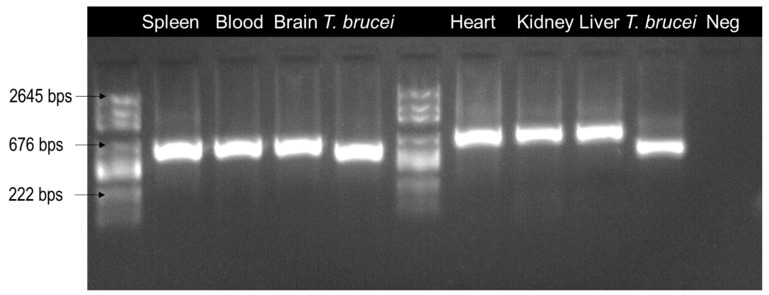
Agarose gel electrophoresis image showing the results of the standard PCR. All organs were positive and the bands correspond to ~550 bps length, except for the positive control used (Trypanosoma brucei). Neg = Negative control.

All organs and blood were positive in qPCR with C_t_ (16–26.5) (Table 1) and in PCR (Figure 2). In the blast analysis, the sequences obtained showed 99.33% identity and 100% cover with *Trypanosoma congolense* riverine/forest-type (acc No. U22319) [18,19].

### 2.4. Further Investigations

Another dog that completed the same mission and returned to France at the same time as the sick dog, as well as six other dogs that spent four months in Côte d’Ivoire around the time of the sick dog, were tested by PCR for the presence of *Trypanosoma* spp. All these samples were negative.

## 3. Discussion

Could such an imported clinical case contaminate other MWD in the army kennel upon its return, and subsequently the livestock or pets surrounding it? This risk assessment justifies our discussion, especially because African trypanosomes represent pathogenic exotic agents in Metropolitan France [20]. African trypanosomosis does not exist in the territory except imported cases and livestock, pets and wildlife animals in France could be sensitive. Several dozen French military dogs are projected annually in areas where CAT is endemic. They return to France after a period of few days to several months (four most often), in their original kennels, located all over France, with a majority of them in Suippes (Marne, East of France) at the 132^e^ Cynotechnical Infantry Regiment. The CAT incubation period is variable. In the case of trypanosomosis due to *T. congolense*, for which dogs are highly sensitive, the incubation period varies from one to three weeks [8], but may last longer (in our case, it developed the disease at least five to eight weeks after his infection in Abidjan).

The creation of a secondary focus requires the establishment of three pillars: the imported parasite, the vectors and the susceptible hosts. Once the pathogen is imported, its spread will be the second step, leading to the creation of an endemic outbreak. This requires early detection and limitation of spread [20].

We hypothesize that this MWD is a reservoir of infective trypanosomes. Despite the consequent number of MWD movements towards Côte d’Ivoire (about 24 per year), our fatal case constitutes an exception. The last confirmed cases among MWD dates back to the years 2001–2002 [16,17]. During that episode, 19 MWD had been affected with *T. congolense*, five died: three of them despite the specific treatment administered on the spot and two on their return to France. All of them were post-mortem diagnosed (hematologic and histologic analyses). The remaining fourteen were successfully treated, seven on the spot and seven after their return to France. The molecule chosen was isometamidium chloride of 1 mg/kg (2% solution or 20 mg/L) [21]. Subsequently to this episode, CAT prevention was systematically administered for the canine military units. The dogs were injected with isometamidium chloride from the first days of their arrival in Côte d’Ivoire and every two months throughout the mission. Since these measures, and up until our dog, no other MWD had developed the disease (over 400 dogs) [17]. Otherwise, any military dog returning from a mission outside mainland France has been subjected to a quarantine period of at least 21 days. During these three weeks, the dog is hosted in the isolation kennel yard, and never leaves its unit. It is observed every day. At the end of the quarantine, the animal is presented to a military veterinarian and blood tests are performed for exotic diseases (ehrlichiosis, Lyme disease, anaplasmosis, heartworm disease and leishmaniosis). A blood smear is also performed to look for trypanosomes.

Further examinations for CAT diagnosis are very important as their clinical picture is not pathognomonic. For *T. congolense*, the dog is particularly more sensitive than other species, and develops an acute clinical form that can be fatal with a devastating neurological picture, as in our case [8]. It occurs especially when the dog is naive for the parasite. Any individual, human or animal, regularly exposed to trypanosomes, seems to develop a trypanotolerance [22,23]. The first signs could be just abatement with anorexia and hyperthermia. The evolution can be very rapid, involving different organs with various symptoms: gastro-intestinal and skin disorders, effusions and oedemas, hemorragic and nervous manifestations. Other sensitive animals express less severe clinical forms. Consequently, CAT clinical diagnosis remains difficult [8,9].

The early detection of CAT requires the examination of blood samples. Blood smear coloration is the common test performed by the Military Veterinary Groups. Its low sensibility has led to a preference for the centrifugation technique on capillary tubes. This procedure makes it possible to detect even small infections (the detection limit is around 500 trypanosomes/mL of blood), mostly six to ten days before the parasite detection by direct blood examinations. In case of suspicion or if the CAT must be included in a differential diagnosis when clinical signs are not very evocative, and in the context of recent stay in Côte d’Ivoire, the use of a laboratory reference test such as PCR is therefore systematic [19].

The introduction of the parasite by the MWD is surveyed, thanks to well-known and well-established procedures, but it remains impossible to completely prevent all events. However, this surveillance can fail, as showed in this case. One scenario previously reported is that of dromedaries imported for breeding purposes in Aveyron (South of France), from the Canary Islands (Spain, 1995 and 2006). This led to a fatal case of *T. evansi* trypanosomosis in 2007, with the detection of antibodies in two of the imported camels and in three others belonging to the farm for years and in sheep from the same area. This focus did not spread to the entire farm, thanks to the elimination of all positive animals [24].

Disease spread requires the presence of competent vectors, mainly haematophagous arthropods. Animal African trypanosomosis are mainly transmitted by tsetse flies absent in France, or, in the case of their accidental importation, they cannot survive enough to eat and reproduce in the French climate. Tsetse flies live at temperatures between 20 °C and 30 °C, in high hygrometry level and shady atmospheres, with vegetation as high as possible. Whenever possible, these Diptera flee areas of human disturbance and settle on the leaves at a maximum height of 50 cm. Any excessive climate change is quickly unfavorable regardless of their development stage and can even be fatal. These factors explain the strict limits of the geographical distribution of *Glossina* [2,25,26]. In France, however, the main risk of trypanosomosis transmission is due to bites from the Tabanidae and Stomoxidae [3,4]. Animal trypanosomosis exists in Europe: *T. theileri* in cattle (transmission by Tabanidae, not very pathogenic). Trypanosomes of birds or amphibians also exist [2].

Among Tabanidae, only females are hematophagous, males feed on nectar. They are diurnal, but only during the warm season. They are found in intensive farming regions and wooded areas and they can fly for long distances (several kilometers). Tabanids bite by tearing off a piece of tissue. The blood flows and the females easily take the blood meal. Many pathogen agents for humans or animals can be transmitted by tabanids, including virus, bacteria, protozoa and helminthes, causing a panel of diseases such as, for instance, pasteurellosis, tularemia, anthrax, leucosis or equine infectious anemia. They also have a direct pathogenic role through their decomposing action on the epidermis of livestock, especially if they are present in large numbers. Their bite is very painful and has a spoliative effect leading to stunted growth and reduced milk production. These biting flies can therefore play a role in the vector transmission of CAT in Metropolitan France [27].

Stomoxidae are small flies that look like house flies. They parasitize cows, horses, and other livestock animals. They are common in stables and sheepfolds, where they find feces to lay their eggs. The difference from other blood-sucking insects is that males also need blood for their biological cycle. They are also vectors for several diseases: equine infectious anemia, African swine fever, West Nile and Rift Valley fever viruses, bacteria and parasites. In addition, Stomoxidae are very often resistant to available insecticides. Here again, their action as vectors for trypanosomosis in France is possible [3,28].

However, such a vector role should be limited by an external parameter: MWD receive, throughout the year, a special prevention against flies, and external anti-parasitics of topical use such as permethrin, fipronil or imidacloprid. Tabanidae and Stomoxidae, therefore, certainly do not bite these dogs. Transmission through infected syringes is unlikely, thanks to good practice in veterinary consultations [17]. Although the probability of importing a case of *T. congolense* into France is low and its spread by local vectors is unlikely, many animal species in metropolitan France are susceptible to trypanosomosis (cattle, suidae, goats, equine and carnivores). Livestock and wildlife animals would be concerned. Clinical disparities exist and have been developed above. In cattle, the parasitaemia remains too low, allowing mechanical transmission of the vector. These animals are often epidemiological dead ends.

## 4. Conclusions

Preventing the creation of a secondary trypanosomosis outbreak in France, in case of reimportation of MWD, is the collective work of military veterinarians, kennel managers and dog handlers. They alert the health services immediately when a dog presents deteriorated general conditions and systematically after quarantine, upon return from the tropical zones. The case reported here allows all the French military veterinarians to be informed about CAT, their clinical picture entering into a consequent differential diagnosis, and to check blood smear screening procedures. The probability of trypanosomosis spread is very low given the deficiencies of the parasite chain at each link: there are few cases of importation, seasonal and regionalized vectors in France, and military working dogs are treated against them. For these reasons, trypanosome transmission in France remains a negligible risk. On the other hand, it seems reasonable to consider the risk of new threats to animal health. They are becoming possible because of globalization, the movement of products, animals and humans, climate change and changes in the ecosystem that may become favorable to some pathogen vectors. Combined measures for the prevention of infectious diseases (vaccination, chemoprophylaxis, vector control, deworming, quarantine, serology or detection of pathogens or antigens, and clinical monitoring upon return) are essential to control the risks of importation of many infectious diseases by animals travelling between endemic areas and their country of origin.

## Figures and Tables

**Table 1:** *Trypanosoma congolense* qPCR results relating to the sick dog on the day of his death. All organ and blood samples were positive.

Samples	C_t_
Spleen	24.14
Blood	16
Brain	18.56
Heart	16.12
Kidney	26.51
Liver	25.77
Pos Ctrl	15.47
Neg Ctrl	N/A
